# Gamma-Glutamylpolyamine Synthetase GlnA3 Is Involved in the First Step of Polyamine Degradation Pathway in *Streptomyces coelicolor* M145

**DOI:** 10.3389/fmicb.2017.00726

**Published:** 2017-04-25

**Authors:** Sergii Krysenko, Nicole Okoniewski, Andreas Kulik, Arne Matthews, Jan Grimpo, Wolfgang Wohlleben, Agnieszka Bera

**Affiliations:** Interfaculty Institute of Microbiology and Infection Medicine Tübingen, Department of Microbiology and Biotechnology, University of TübingenTübingen, Germany

**Keywords:** nitrogen assimilation, *Streptomyces coelicolor*, GS-like enzyme, GlnA3, polyamine utilization, gamma-glutamylpolyamine synthetase

## Abstract

*Streptomyces coelicolor* M145 was shown to be able to grow in the presence of high concentrations of polyamines, such as putrescine, cadaverine, spermidine, or spermine, as a sole nitrogen source. However, hardly anything is known about polyamine utilization and its regulation in streptomycetes. In this study, we demonstrated that only one of the three proteins annotated as glutamine synthetase-like protein, GlnA3 (SCO6962), was involved in the catabolism of polyamines. Transcriptional analysis revealed that the expression of *glnA3* was strongly induced by exogenous polyamines and repressed in the presence of ammonium. The Δ*glnA3* mutant was shown to be unable to grow on defined Evans agar supplemented with putrescine, cadaverine, spermidine, and spermine as sole nitrogen source. HPLC analysis demonstrated that the Δ*glnA3* mutant accumulated polyamines intracellularly, but was unable to degrade them. In a rich complex medium supplemented with a mixture of the four different polyamines, the Δ*glnA3* mutant grew poorly showing abnormal mycelium morphology and decreased life span in comparison to the parental strain. These observations indicated that the accumulation of polyamines was toxic for the cell. An *in silico* analysis of the GlnA3 protein model suggested that it might act as a gamma-glutamylpolyamine synthetase catalyzing the first step of polyamine degradation. GlnA3-catalyzed glutamylation of putrescine was confirmed in an enzymatic *in vitro* assay and the GlnA3 reaction product, gamma-glutamylputrescine, was detected by HPLC/ESI-MS. In this work, the first step of polyamine utilization in *S. coelicolor* has been elucidated and the putative polyamine utilization pathway has been deduced based on the sequence similarity and transcriptional analysis of homologous genes expressed in the presence of polyamines.

## Introduction

*Streptomyces coelicolor* is a Gram-positive, non-motile, soil-dwelling bacterium that belongs to genus *Streptomyces*, phylum Actinobacteria. It can assimilate nitrogen from a variety of mineral (e.g., ammonium, nitrate, and nitrite) and organic sources (e.g., urea, amino acids, peptides, and amino sugars). However, nitrogen sources such as polyamines, which are also omnipresent in nature, have never been reported to be utilized by *S. coelicolor*. Polyamines are widespread in all life kingdoms. Common polyamines include cadaverine, putrescine, spermidine, and spermine. Polyamines can be synthesized *de novo* or taken up from the environment. They are predominantly derived from the following amino acids: ornithine, arginine, and lysine (Kusano and Suzuki, 2015; Miller-Fleming et al., 2015). The canonical biosynthesis pathway begins with decarboxylation of either ornithine or arginine to form putrescine or decarboxylation of lysine to generate cadaverine (Kusano and Suzuki, 2015; Miller-Fleming et al., 2015). Putrescine serves as a substrate for spermine and spermidine biosynthesis via addition of aminopropyl groups donated by the decarboxylated *S*-adenosylmethionine (Kusano and Suzuki, 2015; Miller-Fleming et al., 2015).

Polyamines have been implicated in a wide range of biological processes, thus their intracellular concentration as well as metabolism is tightly regulated (Miller-Fleming et al., 2015). Extensive studies have demonstrated that intracellular polyamine concentrations accumulate during exposure to several stress conditions (reviewed in Miller-Fleming et al., 2015). In bacterial cells, the role of polyamines is less clear and depends on ecological and physiological context. In bacteria, some studies suggest that polyamines might be involved in cell growth stimulation (Yoshida et al., 2004; Igarashi and Kashiwagi, 2006), biofilm formation (Wortham et al., 2007; Lee et al., 2009; Nesse et al., 2015) as well as regulation of translation (Algranati et al., 1976; Nastri et al., 1993; Higashi et al., 2006; Terui et al., 2012) and transcription (Jain and Tyagi, 1987; Huang et al., 1990; Sarkar et al., 1995; Chattopadhyay et al., 2015). Some studies also suggest that they are involved in acid resistance (Jung and Kim, 2003a; Chattopadhyay and Tabor, 2013; Schneider et al., 2013) and the response to oxidative stress (Chattopadhyay et al., 2003; Jung and Kim, 2003b; Tkachenko and Nesterova, 2003; Sakamoto et al., 2015), biosynthesis of siderophores (Griffiths et al., 1984; Brickman and Armstrong, 1996; Oves-Costales et al., 2008; Burrell et al., 2012), SOS system activation (Oh and Kim, 1999) and antibiotic resistance (Nastri and Algranati, 1996; Kwon and Lu, 2006; Tkachenko et al., 2006; Sarathy et al., 2013).

In eukaryotes, polyamines have been described to be implicated in cell growth and development (Pirnes-Karhu et al., 2015; Reis et al., 2016), maturation (Zhou et al., 2009; Tsaniklidis et al., 2016) and proliferation (Heby, 1981; Dudkowska et al., 2003; Maeda et al., 2006; Bercovich et al., 2011; Weiger and Hermann, 2014). They were also reported to be involved in environmental stress response (Valdes-Santiago and Ruiz-Herrera, 2013; Alcazar and Tiburcio, 2014; Minocha et al., 2014; Liu et al., 2015; Pal et al., 2015), defense response (Walters, 2003; Wimalasekera et al., 2011; Zeier, 2013; Hatmi et al., 2014; Mo et al., 2015; Rossi et al., 2015) and apoptosis (Moschou and Roubelakis-Angelakis, 2014; Parhamifar et al., 2014; Cai et al., 2015). Polyamines are positively charged molecules able to interact with negatively charged molecules like RNA, DNA, proteins, polyphosphate, and phospholipids (Norris et al., 2015). Consequently, an imbalance in polyamine metabolism can deeply affect cellular homeostasis. Abolished polyamine biosynthesis leads to a reduction in growth rate in *E. coli*. (Cunningham-Rundles and Maas, 1975) and to complete arrest of cell proliferation in eukaryotic cells (Pitkin and Davis, 1990; Ray et al., 1999; Chattopadhyay et al., 2002; Odenlund et al., 2009). Polyamine excess is toxic for prokaryotic and eukaryotic organisms (Davis and Ristow, 1991; Tome et al., 1997; Pegg, 2013; Miller-Fleming et al., 2015), hence polyamine uptake and utilization as well as biosynthesis are under strict regulation (Kusano and Suzuki, 2015; Miller-Fleming et al., 2015).

**FIGURE 1:**
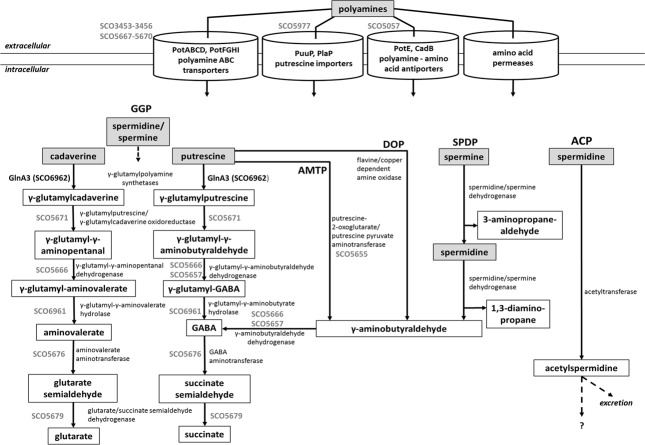
**Combined model of polyamine utilization pathways within prokaryotes based on published studies (Yao et al., 2011; Schneider and Reitzer, 2012; Foster et al., 2013; Campilongo et al., 2014; Kusano and Suzuki, 2015).** GGP, gamma-glutamylation pathway; AMTP, aminotransferase pathway; DOP, direct oxidation pathway; SPDP, spermine/spermidine dehydrogenase pathway; ACP, acetylation pathway. Dashed arrows represent not entirely investigated metabolic pathways. In gray, deduced steps of the polyamine degradation pathway in *S. coelicolor*.

Although polyamine biosynthesis has been broadly studied in evolutionary diverse bacteria and archaea (Tabor and Tabor, 1985; Sekowska et al., 1998; Lee et al., 2009; Nguyen et al., 2015), polyamine catabolism has been mostly studied in gram negative bacteria (Foster et al., 2013; Kusano and Suzuki, 2015). Polyamine utilization pathway is not universal for all bacteria. The currently known polyamine degradative pathways in prokaryotes are summarized in **Figure 1**. These include the γ-glutamylation pathway, aminotransferase pathway, spermine/spermidine dehydrogenase pathway, direct oxidation pathway and acetylation pathway (**Figure 1**). However, concerning the acetylation pathway it remains unclear whether acetylated polyamines are further metabolized or excreted (Forouhar et al., 2005; Bai et al., 2011; Planet et al., 2013; Campilongo et al., 2014; Nguyen et al., 2015). Gram-negative bacteria such as *E. coli* and *P. aeruginosa* degrade putrescine via GABA to succinate through two pathways: the γ-glutamylation pathway (Kurihara et al., 2005; Yao et al., 2011; Schneider and Reitzer, 2012) and the aminotransferase pathway (Shaibe et al., 1985; Schneider and Reitzer, 2012) (**Figure 1**). The initial step of the γ-glutamylation pathway involves the glutamylation of putrescine by the γ-glutamylpolyamine synthetase (PuuA) first reported in *E. coli* (Kurihara et al., 2005). Remarkably, *P. aeruginosa* possess expanded polyamine catabolic pathway involving seven related γ-glutamylpolyamine synthetases (PauA1–PauA7), each specific for polyamines, monoamines, or other substrates (Yao et al., 2011).

Interestingly, the reaction catalyzed by γ-glutamylpolyamine synthetase is comparable to the reaction catalyzed by glutamine synthetase (GS). Both enzymes are able to ligate L-glutamate with an amino group of the specific substrate (polyamine or ammonium, respectively) using energy from ATP. Moreover, both enzyme types share structural similarities (Ladner et al., 2012). Consequently, some proteins annotated as glutamine synthetase-like enzymes (GS-like) may represent still not recognized γ-glutamylpolyamine synthetases. Indeed, *Streptomyces coelicolor* A3(2) harbors two genes, *glnA* (*SCO2198*) and *glnII* (*SCO2210*), encoding GSI and GSII, whose function and regulation was extensively studied (Hillemann et al., 1993; Fink et al., 1999; Weisschuh et al., 2000) as well as three other genes, *glnA2* (*SCO2241*), *glnA3* (*SCO6962*), and *glnA4* (*SCO1613*), annotated as encoding putative GS-like enzymes (Rexer et al., 2006). *In silico* analysis of all *glnA*-like genes: *glnA2*, *glnA3*, and *glnA4* across the actinobacterial genomes revealed that *glnA* has evolved to specialized *glnA*-like genes encoding proteins that might be involved in the colonization and survival in many diverse habitats (Hayward et al., 2009). Distinct ecological niches occupied by actinobacteria exerted specific evolutional pressure on GS genes resulting in diverse, but so far uncharacterized genes (Hayward et al., 2009). Actinobacterial GSI-like enzymes, whose function is unknown (Rexer et al., 2006) were reported to be poorly expressed and their presence in the cell was judged as not essential for bacterial homeostasis at least under laboratory conditions (Harth et al., 2005). In this work, we demonstrated the involvement of the GSI-like enzyme – GlnA3, a gamma-glutamylpolyamine synthetase, in the polyamine degradation in *S. coelicolor* M145.

## Materials and Methods

### Strains and Growth Conditions

*Streptomyces coelicolor* M145 (parental strain) and mutants were cultured for 4–6 days at 30°C on the defined Evans-agar base (modified after Evans et al., 1970) supplemented with different nitrogen sources: 50 mM monosodium glutamate, L-glutamine, ammonium chloride, sodium nitrate, urea, cadaverine dihydrochloride, putrescine dihydrochloride, spermidine trihydrochloride and spermine tetrahydrochloride in appropriate concentrations. Growth experiments in liquid culture were performed using either complex S-medium (Okanishi et al., 1974) or YEME:TSB (1:1) (Kieser et al., 2000) and chemically defined medium Evans-medium (modified after Evans et al., 1970). If appropriate, media were supplemented with polyamines or ammonium chloride as a sole nitrogen source. Strains were cultivated at 30°C, on the rotary shaker (180 rpm) for 4–6 days. Genetic manipulation of *S. coelicolor* M145 was performed as described by Kieser et al. (2000) and Gust et al. (2003). For preparation of the genomic DNA, *S. coelicolor* M145 was grown for 4 days in S-medium and DNA was isolated with the NucleoSpin Tissue Kit (Macherey-Nagel). All strains and plasmids used in this study are listed in the **Table 1**.

**Table 1:** Strains and plasmids used in this study.

Strains	Genotype	Reference
*E. coli* BW25113	K-12 derivative (Δ*araBAD* Δ*rhaBAD*) carrying plasmid pIJ790, Cm^R^	Datsenko and Wanner, 2000
*E. coli ET12567*	*dam*-13::Tn9*, dcm-6, hsdM, Cm^R^*, carrying helper plasmid *pUZ8002*	MacNeil et al., 1992
*E. coli XL1-Blue*	*rec*A1, *end*A1 *gyr*A96*, thi*-1, *hsd*R17, *sup*E44, *rel*A1, *lac* [F′ *pro*AB *lac*IqZΔM15 Tn10 Tet^R^]	Bullock et al., 1987
*S. coelicolor* M145	*S. coelicolor A3(2)* without native plasmids: *spc1^-^* and *spc2^-^*	Kieser et al., 2000
*S. coelicolor* M145 Δ*glnA*	*glnA* mutant strain of *S. coelicolor M145; glnA* replaced by an *aac(3)IV* cassette, Apr^R^	Fink et al., 1999
*S. coelicolor* M145 Δ*glnII*	*glnII* mutant strain of *S. coelicolor* M145; *glnII* replaced by an *aac*(3)IV cassette, Apr^R^	Tiffert et al., 2011
*S. coelicolor* M145 Δ*glnAΔglnII*	*glnAglnII* double deletion mutant strain of *S. coelicolor* M145; Apr^R^ and Hyg^R^	This work
*S. coelicolor* M145 Δ*glnA2*	*glnA2* mutant strain of *S. coelicolor* M145; insertional inactivation of *glnA2* by an *aac*(3)IV cassette, Apr^R^	Nentwich, 2010
*S. coelicolor* M145 Δ*glnA3*	*glnA3* mutant strain of *S. coelicolor* M145; insertional inactivation of *glnA3* by an *aac*(3)IV cassette, Apr^R^	Rexer et al., 2006
*S. coelicolor* M145 Δ*glnA4*	*glnA4* mutant strain of *S. coelicolor* M145; *glnA4* replaced by an *aac*(3)IV cassette, Apr^R^	This work
*S. coelicolor* M145 Δ*glnA3pRM4glnA3*	Complemented *glnA3* mutant strain of *S. coelicolor* M145; Apr^R^ and Km^R^	This work
pRM4	pSET152*ermE*p^∗^ with artificial RBS, Apr^R^	Menges et al., 2007
pIJ10700	pBluescript II KS(+) containing *hyg-oriT* cassette, Hyg^R^	Gust et al., 2003
pJOE2775	pBR322-derived vector with P*rha* expression cassette	Volff et al., 1996
cosmid St3H12	Amp^R^, Kan^R^	Redenbach et al., 1996

### Construction of the Δ*glnA3* Complementation Mutant

To perform a complementation of the mutant, *glnA3* gene without its native promoter was amplified by PCR using C*gln*A3f and C*gln*A3r primers (**Table 2**) and cloned into the multiple cloning site of pRM4 plasmid. The correct construct was confirmed by sequencing and subsequently introduced into the Δ*glnA3* mutant by conjugation using the *E. coli* S17 strain. Clones were then selected on resistant phenotype against kanamycin and apramycin. The correct integration of the pRM4-*glnA3* was confirmed by PCR and sequencing.

**Table 2:** Oligonucleotides used in this study.

Oligonucleotides	Sequences 5′–3′	Reference
RT_*hrdB*1 (control)	GAGTCCGTCTCTGTCATGGCG	This work
RT_*hrdB*2 (control)	TCGTCCTCGTCGGACAGCACG	This work
RT_*glnA3*F	CAGGTGGAGCTGAGCGACTG	Nentwich, 2010
RT_*glnA3*R	AGGCGGAGAGGTGGAGGTG	Nentwich, 2010
RT3453F	CTGGCAGTGACCACCGTTCT	This work
RT3453R	TGTCGGGCAGGTCCAGTTCT	This work
RT3456F	ACCCTGGGCAACACCAGCTATC	This work
RT3456R	AAGTGTCGTCTCCGGCGTTGTC	This work
RT5057F	CGTCTACGGGCTGCTGTTCATC	This work
RT5057R	GTAGTCCAGCATCGCCATCCAC	This work
RT5651F	CAACGCGACGCCGAACTACTAC	This work
RT5651R	GTCAACAAGGGCGATTCGGGAC	This work
RT5655F	CAACGGCTTCTTCTACGG	This work
RT5655R	CAGGATCTGCTCGATCTC	This work
RT5656F	CCATCATCGAGCAGCTCCAG	This work
RT5656R	CAGACGACCTCCACCATCAG	This work
RT5657F	GAAGGCCCTGCTGAAGATCG	This work
RT5657R	CGGCCATCATCATCGGGTAG	This work
RT5658F	TCAGCCCGTCCCTGATGAAC	This work
RT5658R	TCGCAGATCCGGTGGAAGTC	This work
RT5666F	AACTACCCGCTCCAGATG	Thiswork
RT5666R	GCTGACGACGTTGATCAC	This work
RT5667F	TCACGTACACCGAGGACATC	This work
RT5667R	CCAGCGCCTTCTTGTTGTAG	This work
RT5671F	GCGCCAACCAGTTCCACTAC	This work
RT5671R	GACAGCAGGTCGAGCATCAC	This work
RT5676F	CTTCACCCACACCTGTTTC	This work
RT5676R	TGCTTGTACGGCATGTTC	This work
RT5679F	AGAACGGCAAGCCCGTCAAG	This work
RT5679R	TTGTGGGCGCACAGGTTGAG	This work
RT5977F	TTCCAGGACGGCAACCTCAC	This work
RT5977R	AAGACCACGCCGACGATCAG	This work
RT6960F	ATGCCGATCCTGTACGTC	Thiswork
RT6960R	CGAGGTCCTTCTCCAGATG	This work
RT6961F	TATCTCTGCGCGGTCTTC	This work
RT6961R	AACTCGGCCAGTCCATAG	This work
His-*glnA3*F	ACATATGCATCATCATCATCATCATAGCGAGAGCGACCCCGTGCC	This work
His-*glnA3*R	AAAGCTTTCAGTACTTCCAGCGGTACG	This work
*glnII*_hygr_P1	ACGTGCGCCGAGACCCACCCCGAAGGATGTGGCCCCGTGATTCCGGGGATCCGTCGACC	This work
*glnII*_hygr_P2	CCACTGACAACGCGGCCGGGCGGCGGTGGCCCGGGCTCATGTAGGCTGGAGCTGCTTC	This work
C*gln*A3f	ACGAATTCCCGGACGGCACAATG	This work
C*gln*A3r	AGAAAGCTTGCAGGGCCTCGAAAC	This work

### Construction of Δ*glnA*Δ*glnII* Mutant

REDIRECT gene-replacement procedure (Gust et al., 2003) was employed to generate in-frame deletion of the *glnII* gene (*SCO2210*) in previously generated Δ*glnA* mutant (Fink et al., 1999). The *glnII* gene on the cosmid St3H12 was replaced by a hygromycin resistance cassette in *E. coli* BW25113. The hygromycin resistance cassette was amplified by PCR using *glnII*_hygr_P1 and *glnII*_hygr_P2 primers. The obtained cosmid St3H12Δ*glnII* was then transferred into *E. coli* ET12567/pUZ8002 by electroporation and subsequently transferred into *S. coelicolor* M145 Δ*glnA* by conjugation. Hygromycin and apramycin resistant and kanamycin sensitive *trans*-conjugants were isolated from MS-plates supplemented with L-glutamine. Correct marker-less deletion of *glnII* was confirmed by PCR followed by sequencing as well as Southern blot analysis.

### Estimation of the Intracellular or Extracellular Polyamine Level Using HPLC

Level of the intracellular polyamines was analyzed using high-performance liquid chromatography (HPLC), as described by Potter and Paton (2014). The method was modified and optimized for *S. coelicolor*. Cells were harvested by centrifugation (6,000 ×*g*, 10 min at 4°C), washed three times in phosphate-buffered saline (PBS). The 500 mg of the wet cells were resuspended in morpholinepropanesulfonic acid (MOPS) lysis buffer (100 mM MOPS, 50 mM NaCl, 20 mM MgCl2, pH 8.0) and disrupted using glass beads (150–212 μm, Sigma) and a Precellys homogenizer (6500 rpm, 20 – 30 s; Peqlab). Samples were centrifuged (13,000 ×*g*, 10 min at 4°C), the pellet was discarded and supernatant was transferred into new tube. Trichloroacetic acid then was added to a final concentration of 10%, and the mixture was incubated on ice for 5 min. After incubation the mixture was cleared by centrifugation (13,000 ×*g*, 10 min at 4°C), the pH of each sample was neutralized using HCl and the samples were stored at −20°C until analysis. Polyamines were derivatized using pre-column derivatization with *ortho*-phthalaldehyde (OPA)/mercaptoethanol (MCE) (Dr. Maisch GmbH, Ammerbuch). OPA-derivatized polyamines were separated on a Reprosil OPA column (150 mm × 4.0 mm, 3 μm) fitted with a precolumn (10 mm × 4 mm, same stationary phase) (Dr. Maisch GmbH, Ammerbuch) using a HP1090 Liquid Chromatograph equipped with a diode-array detector, a thermostated autosampler and a HP Kayak XM 600 ChemStation (Agilent, Waldbronn). UV detection was performed at 340 nm. The following gradient was used at a flow rate of 1.1 ml/min with solvent A (25 mM sodium phosphate buffer, pH 7.2 containing 0.75% tetrahydrofuran) and solvent B [25 mM sodium phosphate buffer, pH 7.2 (50%), methanol (35%), acetonitrile (15%) by volume]: *t*_0_ = 60% B, *t*_2.5_ = 70%, *t*_12_ = *t*_22_ = 100% B (time in minutes). Cadaverine dihydrochloride, putrescine dihydrochloride, spermidine trihydrochloride, and spermine tetrahydrochloride were purchased from Sigma and used as standards. Standard solutions of polyamines were prepared with distillated water. HPLC detection limit for different polyamines was 0.1 mM.

### Gene Expression Analysis by Reverse Transcriptase/PCR

For the transcriptional analysis experiments, the *S. coelicolor* M145 wild type and the *glnA3* mutant were grown in S-medium. After 4 days, cells were washed twice with defined Evans medium and cultivated for 24 h in defined Evans medium supplemented either with 25 mM ammonium chloride or 25 mM polyamine (cadaverine, spermidine, or putrescine). RNA isolation was performed with an RNeasy kit (Qiagen). RNA preparations were treated twice with DNase (Fermentas). First, an on-column digestion was carried out for 30 min at 24°C, and afterward RNA samples were treated with DNase for 1.5 h at 37°C. RNA concentrations and quality were checked using a NanoDrop ND-1000 spectrophotometer (Thermo Fisher Scientific). cDNA from 3 μg RNA was generated with random nonamer primers (Sigma), reverse transcriptase and cofactors (Fermentas). PCR reactions were performed with the primers listed in **Table 2**. The PCR conditions were 95°C for 5 min; 35 cycles of 95°C for 15 s, 55–60°C for 30 s and 72°C for 30 s; and 72°C for 10 min. As a positive control, cDNA was amplified from the major vegetative sigma factor (*hrdB*) transcript, which is produced constitutively. To exclude DNA contamination, negative controls were carried out by using total RNA as a template for each RT-PCR.

### Survival Assay

The ability of the *S. coelicolor* M145 and Δ*glnA3* mutant to survive in the presence of polyamines was examined by the estimation of the dry weight biomass after 72 and 168 h of incubation in the YEME–TSB (1:1) rich complex medium. Both strains, M145 and Δ*glnA3* mutant were inoculated into YEME–TSB supplemented with putrescine, cadaverine, spermidine as well as spermine (25 mM of each) and incubated for 3 days at 30°C. As a control M145 and Δ*glnA3* mutant were cultivated YEME:TSB without polyamines supplementation and incubated as described above. Samples (1 ml) were taken every day and cells were harvested by centrifugation (16,200 ×*g*, 15 min at 4°C), the residual liquid was removed and pellets were dried for 24 h. The middle value of three technical replicated was compared with the middle value of three biological replicates and the standard error was calculated.

### Light Microscopy and Viability Staining

Morphology and viability of *S. coelicolor* M145 and Δ*glnA3* mutant cells grown in a rich complex YEME–TSB (1:1) medium was analyzed in presence of polyamines. Samples were taken from every culture after 72 and168 h of growth, and obtained perpetrates were observed under phase-contrast microscope under 400× enlargement. To detect live and dead cells, SYTO9 and PI (propidium iodide) stains of the LIVE/DEAD BacLight Bacterial Viability Kit (Molecular Probes) were used. The SYTO 9 green fluorescent stain labels cells with intact membranes, as well as those with damaged ones. PI penetrates cells with damaged membranes, decreasing SYTO 9 stain fluorescence when both dyes are present. Thus, in the presence of both SYTO9 and PI, cells with intact cell membranes appear fluorescent green whereas cells with damaged membranes appear red. The staining solution was prepared by mixing 0.75 μl of component A and B in 500 μl of water. Stained cells were analyzed by the fluorescence microscopy using Olympus BX60 microscope with an Olympus UPlanFl 100 × oil objective and an Olympus BX-FLA reflected light fluorescence attachment. Images were taken with the F-view II camera (Olympus), using TxRed and eGFP filter sets for detection of the fluorescent markers. The ImageJ was used for image processing. Significant number of images (10) was analyzed in a minimum of three independent culture analyses.

### Cloning, Expression, and Purification of His-GlnA3

The gene encoding GlnA3 (SCO6962) was amplified by PCR form *S. coelicolor* genomic DNA and ligated into the expression vector pJOE2775 (Volff et al., 1996) under the control of rhamnose inducible promoter P*rha*. The restriction sites *Nde*I and *Hin*dIII were used for cloning purposes. His-GlnA3 was overexpressed in *E. coli* strain BL21 (DE3) using the auto-induction method for protein production (Li et al., 2011; Studier, 2014). Cells were grown initially at 37°C until the culture density reached an optical density of ∼0.6 at 600 nm at which time the temperature of the shaking incubator was reduced to 20°C. The bacterial cells were then harvested by centrifugation after 48 h cultivation and stored at −20°C until needed. His-GlnA3 was purified by nickel ion affinity chromatography essentially as directed by the resin manufacturer (GE-Healthcare). Purified His-GlnA3 was dialyzed against 20 mM Tris, 100 mM NaCl (pH 8) and immediately used for further analysis.

### GlnA3 *In Vitro* Assay and HPLC/ESI-MS Detection of the Glutamylated-product

HPLC/ESI-MS was used to evaluate putrescine and glutamate as GlnA3 substrates and generated gamma-glutamylated putrescine as a product. Standard reactions typically contained 20 mM HEPES (pH 7.2), 150 mM glutamate sodium monohydrate, 150 mM putrescine dihydrochloride, 20 mM MgCl_2_ × 6H_2_O, and 10 mM ATP were mixed with 10 μg of the purified His-GlnA3 (or without GlnA3 as a control) and incubated at 30°C for 5 min. The reaction was stopped by incubation of the reaction mixture at 100°C for 5 min. HPLC/ESI-MS analysis of the glutamylated product generated by GlnA3 was done on an Agilent 1200 HPLC series using a Reprosil 120 C18 AQ column, 5 μm, 200 mm × 2 mm fitted with a precolumn 10 mm × 2 mm (Dr. Maisch GmbH, Ammerbuch, Germany) coupled to an Agilent LC/MSD Ultra Trap System XCT 6330 (Agilent, Waldbronn, Germany). Analysis was carried out using 0.1% formic acid as solvent A and acetonitrile with 0.06% formic acid as solvent B at a flow rate of 0.4 ml min^−1^. The gradient was as follows: *t*_0_ = *t*_5_ = 0% B, *t*_20_ = 40% B (time in minutes). Injection volume was 2.5 μl, column temperature was 40°C. ESI ionization was done in positive mode with a capillary voltage of 3.5 kV and a drying gas temperature of 350°C.

## Results

### GlnA2, GlnA3, and GlnA4 Annotated as GSI-Like Do Not Have a Glutamine Synthase Function in *S. coelicolor* M145

**FIGURE 2:**
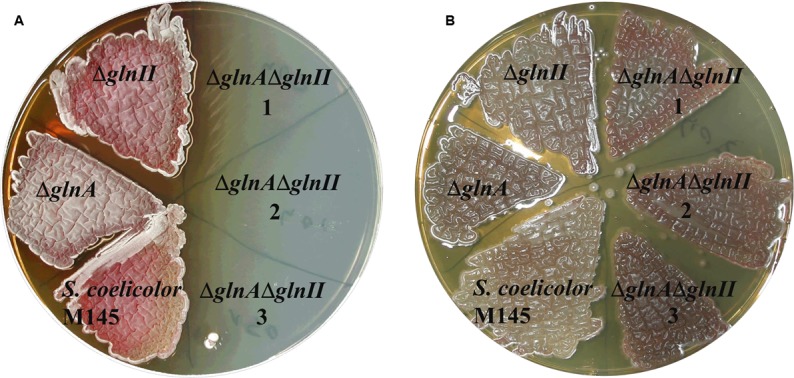
**Phenotypic analysis of parental strain *S. coelicolor* M145 and mutants: Δ*glnA*, Δ*glnII* and Δ*glnA*Δ*glnII* (1, 2, 3 – independent clones) on defined Evans medium supplemented with 50 mM ammonium chloride (A)** or with 50 mM ammonium chloride and 50 mM L-glutamine **(B)**. Deletion of both genes, *glnA* and *glnII*, for GSs, resulted in a glutamine auxotrophic phenotype indicating that GlnA2, GlnA3, and GlnA4 were incapable to substitute GSI and GSII metabolic function in the cell.

In order to determine whether GlnA2, GlnA3, and GlnA4 were involved or not involved in glutamine biosynthesis in *S. coelicolor* M145, single and double mutants of genes *glnA* and *glnII* encoding proven GSs, GSI and GSII, respectively, were deleted in the parental strain M145. REDIRECT gene-replacement procedure (Gust et al., 2003) was employed to delete *glnII* gene (*SCO2210*) in a previously generated Δ*glnA* mutant (Fink et al., 1999) as described in Section “Materials and Methods.” Independent clones of the single Δ*glnA* and Δ*glnII* mutants, of the double Δ*glnAΔglnII* mutant and of the parental strain M145 were tested for their ability to grow on defined Evans-agar supplemented with ammonium chloride (50 mM) as sole nitrogen source. Glutamine synthetases, GSI and GSII catalyze condensation of L-glutamate and ammonia to form L-glutamine in the ATP dependent reaction. Consequently, growth reveals the ability of the strains to assimilate ammonium and to synthesize glutamine whereas absence of growth indicates absence of this ability. *S. coelicolor* M145 and the single deletion mutants, Δ*glnA* and Δ*glnII*, were able to grow on the plates, demonstrating that both *glnA* and *glnII* encode functional GSs as reported previously (Reuther and Wohlleben, 2007). In contrast, the Δ*glnA*Δ*glnII* double mutant was unable to grow on this medium (**Figure 2**) and was auxotrophic for glutamine. Addition of L-glutamine restored growth of the Δ*glnA*Δ*glnII* double mutant on this medium. These results indicated that GlnA2, GlnA3, and GlnA4 do not have a GS function that can substitute for that of GSI and GSII and avoid glutamine auxotrophy.

### The Deletion of *GlnA3*, But Not that of *GlnA2* and *GlnA4* Abolished Polyamine Utilization

**FIGURE 3:**
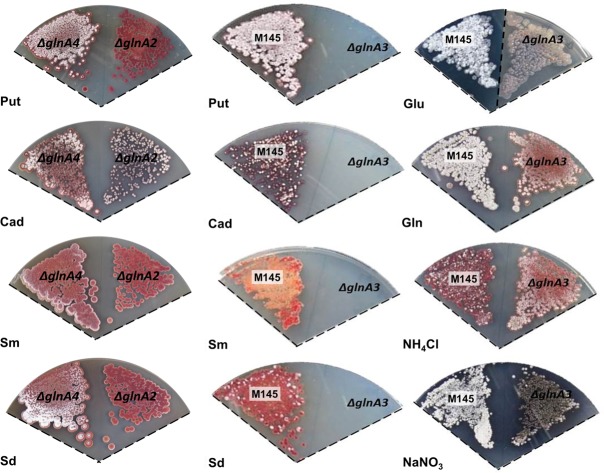
**Physiological role of the *glnA3* gene product in *S. coelicolor* M145 cells grown in the presence of polyamines. (A)** Phenotypic comparison of parental strain *S. coelicolor* M145 and Δ*glnA2*, Δ*glnA3*, and Δ*glnA4* mutants grown on defined Evans medium supplemented with Put – putrescine dihydrochloride (200 mM), Cad – cadaverine dihydrochloride (50 mM), Sm – spermine tetrahydrochloride (25 mM), Sd – spermidine trihydrochloride (25 mM) as well as on Glu – monosodium glutamate (50 mM), Gln – glutamine (50 mM), NH_4_Cl – ammonium chloride (50 mM) and NaNO_3_ – sodium nitrate (50 mM) as sole nitrogen source. Each panel represents observations on a single agar plate, except the phenotypic analysis of the *glnA3* mutant and parental strain in the presence of glutamate has been documented on two separate agar plates as indicated by the dotted line. Deletion of *glnA3* resulted in the no-growth phenotype in the presence of high polyamine concentrations.

In an attempt to decipher the function of GS-like enzymes, the genes *glnA2*, *glnA3*, and *glnA4* were deleted in the parental strain *S. coelicolor* M145. The Δ*glnA2* (Nentwich, 2010), Δ*glnA3* (Rexer et al., 2006), and the Δ*glnA4* (this work) mutants were tested for their ability to utilize different nitrogen sources. Phenotypic analysis was performed on defined Evans-agar supplemented with the following sole nitrogen sources: ammonium chloride, sodium nitrate, monosodium L-glutamate, L-glutamine, putrescine dihydrochloride, cadaverine dihydrochloride, spermidine trihydrochloride, and spermine tetrahydrochloride. Growth and morphology of the parental strain M145 and the Δ*glnA2, ΔglnA3*, and Δ*glnA4* mutants were monitored on defined Evans-agar after 3 to 12 days of incubation at 30°C. Remarkably, *S. coelicolor* M145 could grow on all tested nitrogen sources (**Figure 3**). Interestingly, growth of the *S. coelicolor* M145 on polyamines resulted in delayed aerial mycelium and spore formation as well as abolition of actinorhodin (blue antibiotic) and enhanced prodigiosin (red antibiotic) production (**Figure 3**). Growth of the *glnA2, glnA3*, and *glnA4* mutants was similar to that of the parental strain in the presence of ammonium chloride, sodium nitrate, L-glutamine, and monosodium L-glutamate. Growth of the *glnA3* mutant, but not that of the *glnA2* and *glnA4* mutants, was inhibited in the presence of putrescine, cadaverine, spermidine, and spermine (**Figure 3**). The complementation of the *glnA3* mutant by the *glnA3* gene under control of the constitutive promoter (P*ermE*) restored the growth of this mutant on polyamine plates (Supplementary Figure S1). Altogether these results indicated that the disruption of *glnA3*, but not that of *glnA2* and *glnA4*, completely abolished the utilization of polyamines as a nitrogen source. GlnA2 and GlnA4 have obviously a function different from that of GlnA3 since their deletion did not influence polyamine utilization. Interestingly, the *glnA3* mutant showed defect in formation of aerial mycelium as well as sporulation on defined medium supplemented with glutamate (50 mM). Moreover, enhanced actinorhodin and prodigiosin production was observed on the nitrate, glutamine and ammonium plates, respectively (**Figure 3**), indicating an impact of *glnA3* deletion on the secondary metabolites production and morphological differentiation in *S. coelicolor*.

### Expression of *glnA3* Is Induced by Polyamines and Starvation Conditions

**FIGURE 4:**
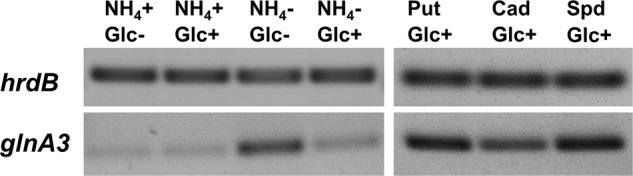
**Transcriptional analysis of *glnA3* in the presence of polyamines and ammonium as a sole nitrogen sources.** Reverse transcriptase/PCR of *glnA3* and *hrdB* (control) from *S. coelicolor* M145 cultivated in defined Evans medium with low (5 mM) or high (50 mM) concentrations of ammonium chloride or polyamines (Put – putrescine; Cad – cadaverine and Spd – spermidine, 25 mM of each) and glucose as a sole carbon source high Glc+ (25 g/l) or low Glc– (2.5 g/l). Total RNA was isolated from mycelium harvested after 24 h of cultivation in the defined Evans medium.

Reverse transcription/PCR experiments were performed as described in Section “Materials and Methods” to determine whether *glnA3* expression was influenced by *N-* and/or *C-*concentrations as reported for *glnA* and *glnII* (Amin et al., 2016) as well as to investigate the expression of *glnA3* in the presence of polyamines. For this purpose, parental strain M145 was grown in complex S-medium subsequently transferred into defined Evans medium supplemented either with polyamines (25 mM) or ammonium chloride (25 mM) as a sole nitrogen source. Total RNA isolated after 24 h from *S. coelicolor* M145 was used to generate cDNA. Subsequently, reverse transcription/PCR analysis was performed using primers internal to *glnA3*. HrdB, encoding the main sigma factor of RNA polymerase, that has relatively constant levels of expression throughout growth (Buttner et al., 1990) was used as internal control. Expression of genes encoding GSs, *glnA* and *glnII*, was shown to be strongly enhanced in condition of ammonium limitation and reduced or completely abolished in condition of ammonium proficiency in *S. coelicolor*, irrespective of the glucose concentration (Amin et al., 2016). Interestingly, the expression of *glnA3* was also strongly enhanced under ammonium limitation, but only in combination with low glucose concentration suggesting that its expression does not require polyamines. Furthermore, transcriptional analysis of *glnA3* revealed strong induction of the expression of this gene in the presence of putrescine, cadaverine and spermidine (**Figure 4**), putative substrates of the encoded enzyme.

### Transcriptional Analysis-Based Identification of Orthologs of the Putative Polyamine Transport and Polyamine Utilization Genes in *S. coelicolor* M145

**FIGURE 5:**
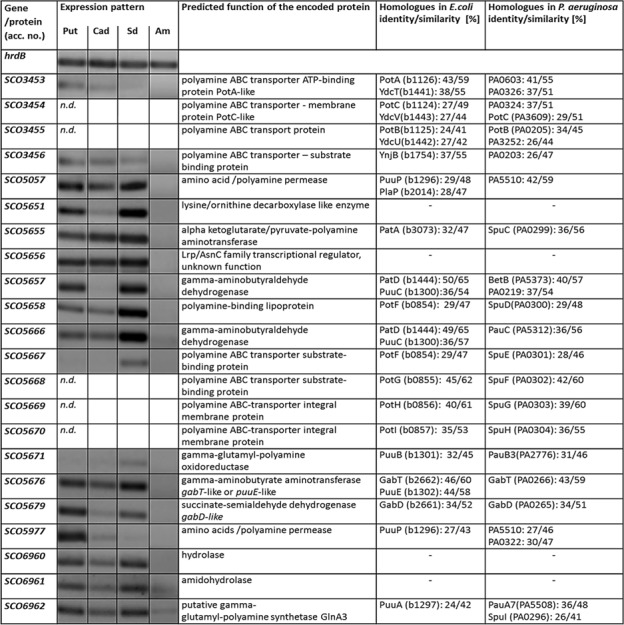
**Transcriptional analyses of putative gene homologs encoding predicted proteins involved in polyamine degradation in *S. coelicolor*.** Reverse transcriptase/PCR of genes and *hrdB* (control) from *S. coelicolor* M145 cultivated in defined Evans medium with polyamines (Put – putrescine; Cad – cadaverine or Spd – spermidine, 25 mM of each) and glucose as a sole carbon source Glc (25 g/l). Total RNA was isolated from mycelium harvested after 24 h of cultivation in the defined Evans medium.

Polyamine uptake and utilization pathways are largely uncharacterized in streptomycetes. BlastP analysis was used to predict uptake systems and putative enzymes involved in polyamine utilization in *S. coelicolor*, whose amino acid sequences are closely related to the amino acid sequences of the characterized proteins from *E. coli* and *P. aeruginosa* (Kusano and Suzuki, 2015). Similarity based prediction revealed 18 strong homologs in *S. coelicolor* (genome accession number AL645882). Their amino acid identities ranged from 24 to 50%, while their similarities ranged from 41 to 65% (**Figure 5**). To demonstrate that these genes were induced by polyamines, their expression patterns were analyzed in the presence of putrescine, cadaverine, and spermidine as sole *N*-source as well as ammonium as control.

Analysis of the *glnA3* genomic region revealed two genes *SCO6960* and *SCO6961* located downstream of *glnA3* and likely co-transcribed with the latter. *SCO6961* encodes an uncharacterized protein belonging to the amidohydrolase 2 family, whereas *SCO6960* encodes a small protein of unknown function conserved among streptomycetes. However, no orthologs of these genes could be found in *E. coli* and *P. aeruginosa*, suggesting that the polyamine utilization pathway might be different in *S. coelicolor*. Transcriptional analysis of the *SCO6960* and *SCO6961* indicated that the expression of these genes was inducible by polyamine as that of *glnA3* (**Figure 5**). In contrast, the expression of the genes *SCO6963, SCO6964*, and *SCO6965* located upstream of this putative operon and encoding uncharacterized lipoprotein and two small proteins of unknown function were not induced in conditions of ammonium limitation nor in the presence of polyamine (data not shown).

Furthermore, BlastP analysis predicted two ABC transporters and two permeases potentially involved in the uptake of polyamines. The *SCO5667-70* and *SCO3453-56* predicted gene products resemble the ABC transport systems PotFGHI or SpuEFGH from *E. coli* and *P. aeruginosa*, respectively. The expression of these genes was only weakly inducible in the presence of polyamines (**Figure 5**). In contrast, the expression of the genes encoding the putative polyamine permeases *SCO5057* and *SCO5977* was strongly induced in the presence of all polyamines or only putrescine, respectively (**Figure 5**). The expression of *SCO5658* (encoding predicted polyamine binding protein) was strongly induced in the presence of spermidine (**Figure 5**).

Prediction of genes encoding enzymes involved in the post-glutamylation steps in *S. coelicolor* revealed orthologs for gamma-glutamylpolyamine oxidoreductase (SCO5671), polyamine-pyruvate/2-oxoglutarate transaminase (SCO5655) as well as close orthologs of (gamma-glutamyl-) gamma-aminobutyraldehyde dehydrogenases PatD and PuuC (SCO5657 and SCO5666) and *SCO5676* encoding predicted GabT-like gamma-aminobutyrate transaminase and finally *SCO5676*, encoding predicted succinate semialdehyde dehydrogenase (GabD-like). The expression of all these genes (except *SCO5671*) was strongly induced in the presence of polyamines (**Figure 5**). Altogether, putative homologs of genes encoding predicted polyamine uptake systems and proteins involved in the polyamine utilization in *S. coelicolor* showed enhanced expression in the presence of polyamines whereas their expression was abolished in the presence of ammonium. To summarize, the search for proteins orthologs of those involved in the catabolism of polyamine in other bacteria using BlastP and the analysis of gene expression patterns, led us to predict, so far unknown, polyamine degradation pathway in *S. coelicolor* (**Figure 1**).

### *GlnA3* Deletion Mutant Accumulates Polyamines Intracellularly

**FIGURE 6:**
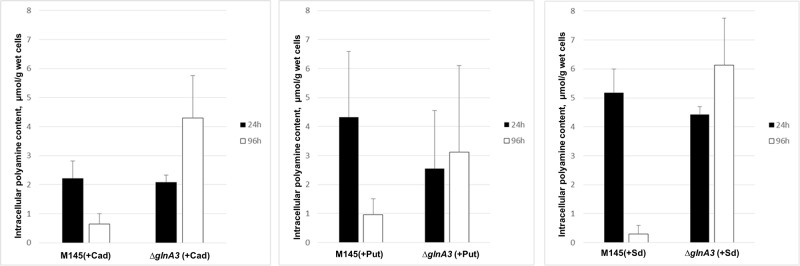
**Effect of *glnA3* deletion on intracellular polyamine concentration.** The intracellular polyamine level of parental strain *S. coelicolor* M145 and Δ*glnA3* mutant was monitored after 24 and 96 h of cultivation in defined Evans medium supplemented with polyamines (Put – putrescine; Cad – cadaverine or Sd – spermidine, 25 mM of each). The mean value of three biological replicates was calculated in μmol per 1 g of wet cells. Error bars indicate standard error of three biological replicates.

**FIGURE 7:**
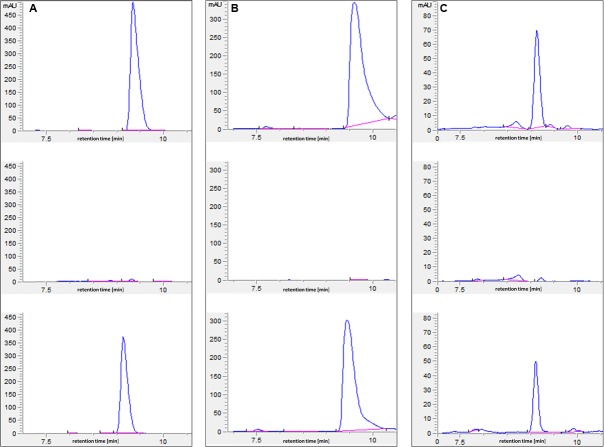
**HPLC-based detection of polyamines in supernatant from the 96 h culture of parental strain M145 and Δ*glnA3* mutant.** Both strains were grown in defined Evans medium supplemented with either **(A)** putrescine (25 mM), **(B)** cadaverine (25 mM) or **(C)** spermidine (25 mM). Upper HPLC chromatogram: polyamine standard, middle HPLC chromatogram: detection of polyamines remained in the supernatant of the M145 culture, lower HPLC chromatogram: detection of polyamines remained in the supernatant of the Δ*glnA3* mutant culture. Results indicate that only parental strain *S. coelicolor* M145 was able to completely utilize whole polyamines from the medium after 96 h of incubation.

In an attempt to understand the basis of polyamine toxicity in the *glnA3* mutant grown in the presence of polyamine, the intracellular polyamine content of *S. coelicolor* M145 and *glnA3* mutant was analyzed by HPLC. To do so, the parental strain M145 and the *glnA3* deletion mutant were cultivated in a complex S-medium for 4 days at 30°C. Cells were harvested and washed with Evans medium without *N*-source to remove remaining complex *N*-sources. Washed cells were transferred into defined Evans medium containing polyamines as a sole *N*-source and incubated at 30°C for further 4 days. Samples for the biomass determination and extraction of intracellular polyamines were collected every day. Cell pellets and supernatants were used to extract intracellular and extracellular polyamines, respectively. Comparison of intracellular polyamine levels of both strains revealed three, six and twenty fold higher levels of putrescine, cadaverine, and spermidine in Δ*glnA3* mutant, respectively (**Figure 6**) than in the parental strain after 4 days of cultivation. The intracellular concentration of polyamines decreased over time in the parental strain M145, but not in the mutant strain. After 4 days of cultivation, intracellular polyamine concentrations ranged between 0.5 and 0.7 μmol/g of biomass, whereas that of the *glnA3* mutant ranged between 3.1 and 5.1 μmol/g. Furthermore, comparison of extracellular polyamine levels in the parental strain culture and *glnA3* culture revealed strong differences. The residual level of polyamines in the *glnA3* mutant culture supernatant remained high from the first to the fourth day of incubation (**Figure 7**), whereas after 4 days of incubation no peak for polyamines could be detected in the culture supernatant of the parental strain. Altogether, these data indicated that the *glnA3* mutant was able to uptake polyamines, but was unable to degrade them. The absence of polyamine degradation in the *glnA3* mutant obviously resulted in high concentration of intracellular polyamine, likely to be responsible for the inhibition of further uptake of external polyamines (**Figure 7**).

### The Δ*glnA3* Mutant Grown in Complex Medium with Polyamines Showed Atypical Mycelial Morphology and Short Lifetime Span

**FIGURE 8:**
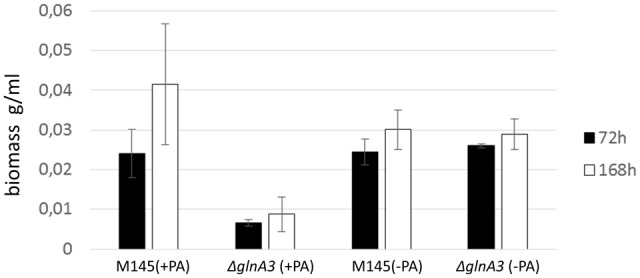
**Effect of the *glnA3* deletion on a biomass accumulation in a rich complex medium supplemented with and without polyamines.** Monitoring of changes in biomass accumulation of the parental strain *S. coelicolor* M145 and Δ*glnA3* mutant in the YEME:TSB (1:1) medium supplemented with polyamines total concentration 100 mM (putrescine, cadaverine, spermidine, and spermine, 25 mM of each) after 72 and 168 h of incubation. Error bars indicate standard error of three biological replicates.

The Δ*glnA3* mutant and its parental strain *S. coelicolor* M145 were cultivated in a rich complex liquid medium (YEME:TSB) supplemented or not (control) with polyamines (putrescine, cadaverine, spermidine, and spermine, 25 mM of each). Biomass yields as well as morphology of both strains were monitored over 7 days. The Δ*glnA3* mutant yielded significantly lower biomass than the parental strain after 72 and 168 h of cultivation in the complex medium supplemented with polyamines (**Figure 8**). Prolonged incubation of the Δ*glnA3* mutant culture did not result in any significant biomass increase. In this medium, *S. coelicolor* M145 showed its typical pellet growth whereas growth of the Δ*glnA3* mutant was dispersed. This indicated that despite the presence of alternative nitrogen sources in this complex medium, besides polyamines, growth of Δ*glnA3* mutant was impaired most likely because of intracellular accumulation of polyamines.

**FIGURE 9:**
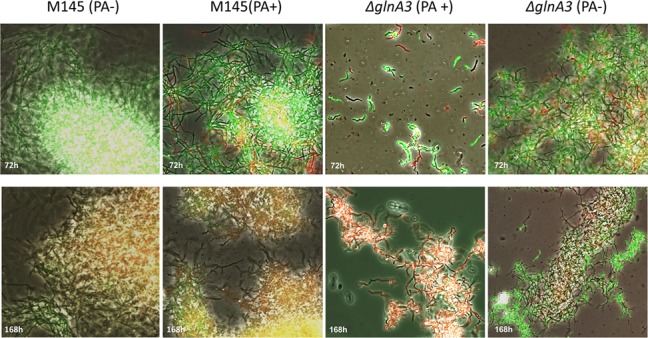
**Effect of the *glnA3* deletion on cell morphology and its viability in the presence of polyamines.** Parental strain *S. coelicolor* M145 and Δ*glnA3* mutant were cultivated in YEME:TSB (1:1) medium with or without polyamines total concentration 100 mM (putrescine, cadaverine, spermidine, and spermine, 25 mM of each). Phase contrast microscopic pictures of parental strain M145 and Δ*glnA3* mutant mycelium stained with SYTO9/PI were taken after 72 and 168 h of growth (under 400× enlargement).

**FIGURE 10:**
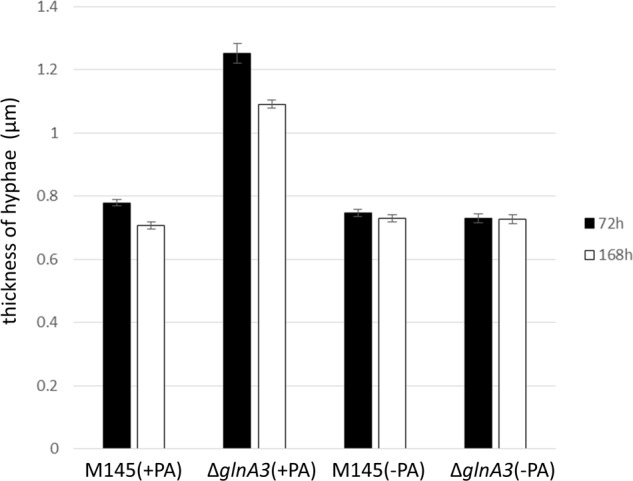
**Comparison of the hyphae thickness of parental strain *S. coelicolor* M145 and Δ*glnA3* mutant grown in the presence of polyamines.** Parental strain *S. coelicolor* M145 and Δ*glnA3* mutant were cultivated in YEME:TSB (1:1) medium with or without polyamines. The hyphae thickness was measured after 72 and 168 h of growth. Error bars indicate standard error of *n* = 100 biological replicates.

The mycelium of the Δ*glnA3* mutant grown in the complex medium with polyamines was observed under microscope. After 3 days of cultivation, the parental strain formed a long and compact, branched mycelium constituting pellets whereas the Δ*glnA3* mutant formed non-branched mycelium (**Figure 9**). The mycelial fragments of the Δ*glnA3* mutant were significantly shorter and thicker than those of the parental strain M145 after 3 or 7 days of cultivation in the medium containing polyamines (**Figure 10**).

In order to assess more precisely the toxic effect of polyamines, the viability of the Δ*glnA3* mutant was assessed with SYTO9/PI staining. The SYTO 9 green fluorescent stain labels all cells, whereas PI penetrates only cells with damaged membranes, decreasing SYTO 9 stain fluorescence when both dyes are present. In the presence of both, SYTO9 and PI, cells with intact cell membranes appear fluorescent green and dead cells with damaged membranes appear red. Whereas the parental strain M145 appeared green (alive) in the presence of polyamines, most filaments in the culture of the Δ*glnA3* mutant were red (dead) after 7 days of incubation (**Figure 9**). These results demonstrated that the absence of GlnA3 led to the toxic accumulation of polyamine that impaired growth and viability and suggested that GlnA3 plays a major role in polyamine degradation.

### *GlnA3* a Predicted Gamma-Glutamylpolyamine Synthetase in *S. coelicolor* M145

The GlnA3 protein belongs to large class of ligases which form carbon-nitrogen bonds using energy from ATP (class 6.3.1). This class contains twenty different subclasses of enzymes including GS (class 6.3.1.2), glutamate – ethylamine ligase (class 6.3.1.6) and glutamate-putrescine ligase (6.3.1.11). GlnA3 protein sequence analysis by InterProScan^1^ predicted two enzymatic domains: N-terminal GS, beta-Grasp domain (IPR008147) and the C-terminal catalytic domain of GS (IPR008146). Searching for functionally and structurally characterized GlnA3 homologs from the Protein Data Bank (PDB) revealed a gamma-glutamylmonoamine/polyamine synthetase PauA7 (PA5508) as a likely. PauA7 is involved in monoamine/polyamine gamma-glutamylation in *P. aeruginosa* (Ladner et al., 2012). The available crystal structure of Pau7 (Protein Data Bank entry: 4HPP) and GS GSI*_St_* from *S. typhimurium* (Protein Data Bank entry: 1FPY) were used as the templates to generate a structural identical conserved binding residues for glutamate and ATP as well as two divalent metal ions (Mg^++^/Mn^++^). However, the binding pocket for ammonium and monoamine differ significantly. The GSI*_St_* (as well as other eukaryotic and bacterial GS) and the PauA7 require two divalent metal ions per one subunit for activity. These ions have a structural as well as a catalytic role. Comparison of the conserved catalytic residues for the binding of divalent metal ions revealed four conserved glutamate residues E151, E153, E207, E214 and one conserved histidine residue H263 in the GlnA3 model structure (corresponding to E131, E133, E180, E187, and H236 in PauA7, as well to E129, E131, E212, E220, and H269 in GSI*_St_*, respectively). Moreover, three conserved residues involved in coordination of the glutamate binding in GSI*_St_* and PauA7, were found in the GlnA3 model structure. These GlnA3 residues (N260, G261, and R316) correspond to the N233, 258 G234, and R290 in PauA7 as well as to N264, G265, and R321 in GSI*_St_*. Finally, two conserved residues (H265 and R339 both corresponding to H271 und R344 in GSI*_St_*) important for coordination of the beta-phosphate and alpha-phosphate group of ADP were found in the GlnA3 model. This comparative *in silico* analysis demonstrates that GlnA3 possess conserved residues for binding of two metal ions, L-glutamate and ATP (Supplementary Figure S2). An essential element of GSI*_St_* catalytic pocket is a loop, termed “the E327 flap” (GSI*_St_*). The GSI active site is located between two subunits, and the E327-flap closes the catalytic site by interaction of the E335 with D50 from an adjacent subunit within the same ring, shielding the γ-glutamyl phosphate intermediate from hydrolysis. The second conserved residue D50 is involved in the deprotonation of ammonium for attack on the γ-glutamyl phosphate. Interestingly, these key acidic residues (E327 and D50) essential for the catalytic synthesis of glutamine in GSI*_St_* are not conserved in PauA7 nor in GlnA3 (Supplementary Figure S2). In PauA7 structure and GlnA3 model these loops are much bigger and instead E327, a non-polar W327 residue occupies analogous position in the GlnA3 model structure (corresponding to W296 in PauA7). The conserved residue D50 that increases the affinity for ammonium binding in GSI*_St_* is replaced by G40 and G63 in PauA7 and 273 GlnA3, respectively. Moreover, the conserved Y179 in GSI*_St_* that coordinates the ammonium binding pocket is substituted by A147 in PauA7 and A169 in GlnA3, providing much more space for a bulky substrate such as polyamine (Supplementary Figure S2). The significant overall similarity of GlnA3 to the gamma-glutamylmonoamine/polyamine synthetase PauA7, the lack of the conserved residues for ammonium binding as well as our observations strongly suggest that GlnA3 may function as a gamma-glutamylpolyamine synthetase catalyzing the first step of polyamine degradation.

### GlnA3 Catalyzes Gamma-Glutamylation of Putrescine *In Vitro*

**FIGURE 11:**
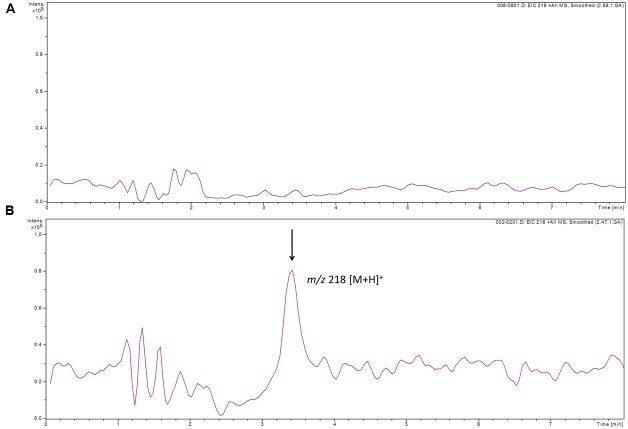
**HPLC/ESI-MS analysis of the glutamylated product generated by GlnA3 in *in vitro* assay.** Two samples were analyzed in the MS positive mode: reaction mixtures without addition of GlnA3 **(A)** and with addition of GlnA3 **(B)**. Extracted ion chromatograms for the GlnA3 reaction product corresponding to gamma-glutamylputrescine with charge to mass ratio of 218 m/z was shown **(B)**, and no product in the sample without addition of GlnA3 could be detected **(A)**.

To determine whether GlnA3 was able to catalyze the predicted glutamylation reaction we developed an HPLC/ESI-MS based assay designed to detect a gamma-glutamyl product formed by GlnA3. For this, GlnA3 was overexpressed in *E. coli* BL21 (DE3) and purified by nickel ion affinity chromatography. The purified His-GlnA3 was used in an *in vitro* assay as described in Section “Materials and Methods.” The reaction conditions were as follows: 10 μg of His-GlnA3 were incubated at 30°C with glutamate, putrescine, MgCl_2_ and ATP in HEPES buffer (pH 7.2). The reaction was stopped after 5 min. by incubation at 100°C for 5 min. HPLC analysis of generated product was performed in a positive MS mode. Result indicated that GlnA3 was able to use glutamate and putrescine as substrates in the ATP dependent reaction, generating a product with the mass to charge ratio of 218 *m/z*, corresponding to calculated mass of gamma-glutamylputrescine, confirming the function of GlnA3 as a gamma-glutamylpolyamine synthetase (**Figure 11**).

## Discussion

In this study, we demonstrated that *S. coelicolor* M145 is able to utilize polyamines such as putrescine, cadaverine, spermidine, and spermine as a sole nitrogen source and it is able to withstand high polyamine concentrations. Our results indicate that this ability is due to GlnA3 since the deletion of *glnA3* resulted in intracellular polyamine accumulation, abnormal morphology of the mycelial fragments as well as a polyamine sensitive phenotype resulting in cell death. Our *in vivo* data concerning the deletion of *glnA* and *glnII* encoding GSs, GSI and GSII, respectively, indicated that GlnA3 doesn’t fulfill a function of GS. Comparative *in silico* analysis of PauA7, GSI and GlnA3 as well as the *in vitro* GlnA3 enzymatic assay revealed that GlnA3 bear features of a gamma-glutamylpolyamine synthetase, an enzyme catalyzing the first step of polyamines catabolism in *Streptomyces*.

Using BlastP for comparative analyses of *Streptomyces* proteome sets (taxid: 1883), 116 homologs of the predicted GlnA3 protein were identified with 75–99% sequence identity and 95–100% query cover in *Streptomyces* sp. Frequent occurrence of GlnA3 homologs in *Streptomyces* genomes as well as its highly conserved protein sequence in all strains indicates its evolutionary importance. Homologs of the predicted GlnA3 protein were also found in other actinobacteria such as: *Nocardia* spp., *Rhodococcus* spp., *Frankia* spp., *Amycolatopsis* spp., *Kitasatospora* spp., *Saccharopolyspora* spp. and *Mycobacterium* spp. (45–76% identity and 92–100% query cover).

Since orthologs of GlnA3 were found also in some pathogenic actinobacteria, GlnA3 might ensure colonization and persistence in the host by conferring resistance against toxic concentrations of polyamines produced during infection. Indeed, the polyamine content was reported to increase dramatically in both proliferating cells and extracellular tissue fluids during inflammation, tissue regeneration and cell damage (Yatin, 2011). Increased polyamines levels have been demonstrated at inflammatory sites of bacterial and viral infections (Hirsch and Dubos, 1952; Colombatto et al., 1989; Clarke and Tyms, 1991; Lasbury et al., 2007). Interestingly, pathogens belonging to the actinobacteria group are able to survive in a tissue in presence of high polyamine concentrations produced by defense system in a response to infection and stress (Liao et al., 2009; Grasemann et al., 2012; Jimenez-Bremont et al., 2014). Symbiotic actinobacteria can survive in the nitrogen fixing nodules where they are constantly confronted with fluctuating polyamine concentrations (Fujihara et al., 1994). Free living *S. coelicolor* and other non-motile actinobacteria might have to cope with high concentration of polyamines that are released during the decomposition of animal cadaver and plant tissues in the soil.

The search for proteins orthologs of those involved in the catabolism of polyamine in other bacteria using BlastP and the analysis of the expression patterns of these genes, led us to predict, polyamine glutamylation pathway in *S. coelicolor* (**Figure 1**). We were able to show that the gamma-glutamylpolyamine synthetase GlnA3 is important for polyamine catabolism in *S. coelicolor* under the conditions tested. This implies that further post-glutamylation steps of the so far known polyamine glutamylation pathway in *E. coli* and *P. aeruginosa* (or a variation of this pathway) are most likely present in streptomycetes. The second step of glutamylation pathway requires a gamma-glutamyl polyamine oxidoreductase (PuuB in *E. coli*/pauB1-B4 in *P. aeruginosa*). The SCO5671 (predicted ortholog of the PuuB) showed no expression in the presence of putrescine and cadaverine, but a weak expression has been observed in the presence of spermidine. It remains to be elucidated whether other potential homologs (SCO1281, SCO6051) with lower sequence similarity might represent gamma-glutamylpolyamine oxidoreductase PuuB in streptomycetes. The next step of the polyamine glutamylation pathway involves predicted PuuC dehydrogenase homologs (SCO5666, SCO5657), followed by the predicted hydrolase SCO6961. Last two steps of polyamine glutamylation pathway lead to the formation of succinate and are catalyzed by the predicted GabT and GabD homologs, SCO5676 and SCO5679, respectively. Interestingly, expression of the *SCO5676* has been shown to be stimulated by arginine (a precursor of putrescine) in *S. coelicolor* M145 (Perez-Redondo et al., 2012).

Moreover, the predicted PatA aminotransferase (SCO5655), along with orthologs of additional predicted aminotransferases and dehydrogenases, suggests the possibility of an alternate pathway in streptomycetes. Interestingly, induction of the SCO5655 has been reported to be stimulated by diamide (Kallifidas et al., 2010), however, not by arginine (Perez-Redondo et al., 2012). Further genetic analysis of SCO5655 and other potential aminotransferase homologs (SCO1223, SCO1284, and SCO6769) would be required to determine whether a PatA-dependent pathway is functional. The regulation and importance of glutamylation and aminotransferase pathways may dependent on polyamine concentration or another environmental parameter such as temperature (Schneider and Reitzer, 2012).

A better knowledge of polyamine utilization in actinobacteria is of fundamental importance, since this pathway may be important for the survival strategy of pathogenic, symbiotic, and non-pathogenic actinobacteria living in as diverse habitats as human, animal, and plant tissues or in soil environment, respectively. Our work constitutes the first attempt to decipher polyamine metabolism in the actinobacterial model organism *Streptomyces coelicolor* via the characterization of a major polyamine resistance factor, the gamma-glutamylpolyamine synthetase GlnA3 that is indispensable for survival under high polyamine concentration.

## Author Contributions

AM performed the reverse transcriptase/PCR analysis. AK performed the HPLC and HPLC/MS analysis. JG constructed and analyzed the Δ*glnA/glnII* mutant. NO constructed the Δ*glnA4* mutant as well as was involved in technical assistance. NO and SK performed phenotypic analysis of all mutants and parental strain M145 on Evans medium with different nitrogen sources. SK performed complementation of the Δ*glnA3* mutant, survival and viability assays, microscopic analysis of cells, intracellular polyamine analysis and bioinformatics analysis of DNA and protein sequences. SK cloned, overexpressed and purified GlnA3 protein and performed GlnA3 *in vitro* assay. AB formulated the original problem and provided direction and guidance as well as designed the study and developed the methodology. AB generated and analyzed the model of GlnA3 structure. WW provided helpful feedback on an early draft of the paper and assisted with data analysis. AB contributed to the writing of the manuscript and resolved final approval of the version to be published.

## Conflict of Interest Statement

The authors declare that the research was conducted in the absence of any commercial or financial relationships that could be construed as a potential conflict of interest.

## Footnotes

1 http://www.ebi.ac.uk/interpro
