# Supplementary material for: Gamma-Glutamylpolyamine Synthetase GlnA3 Is Involved in the First Step of Polyamine Degradation Pathway in Streptomyces coelicolor M145
Source: Front Microbiol. 2017 Apr 25;8:726. doi: 10.3389/fmicb.2017.00726 (PMC5403932; doi:10.3389/fmicb.2017.00726)
Supplement: Supplementary file 1 [file Presentation_1.PPTX]

## Slide 1
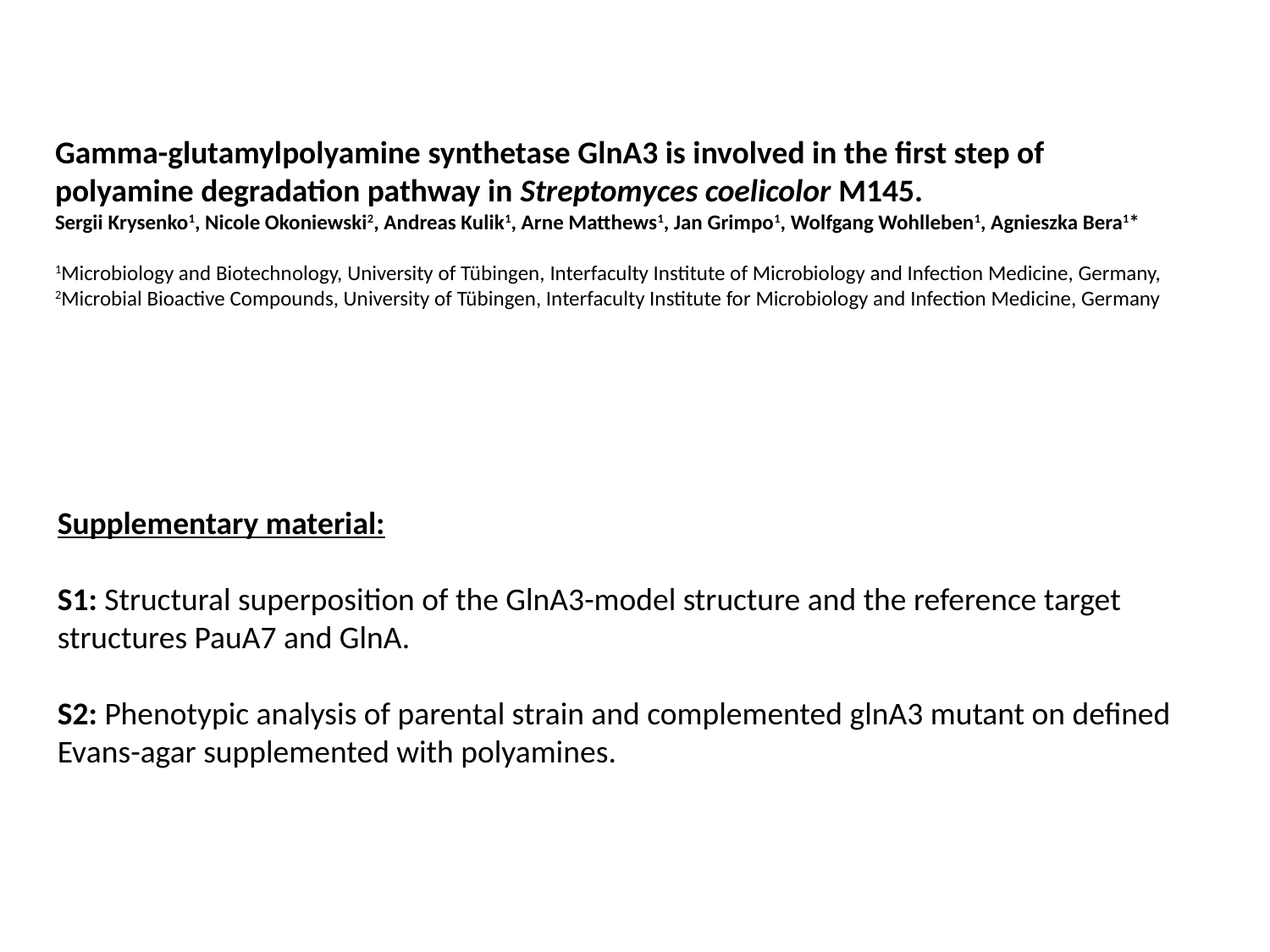

Gamma-glutamylpolyamine synthetase GlnA3 is involved in the first step of
polyamine degradation pathway in Streptomyces coelicolor M145.
Sergii Krysenko1, Nicole Okoniewski2, Andreas Kulik1, Arne Matthews1, Jan Grimpo1, Wolfgang Wohlleben1, Agnieszka Bera1*
1Microbiology and Biotechnology, University of Tübingen, Interfaculty Institute of Microbiology and Infection Medicine, Germany,
2Microbial Bioactive Compounds, University of Tübingen, Interfaculty Institute for Microbiology and Infection Medicine, Germany
Supplementary material:
S1: Structural superposition of the GlnA3-model structure and the reference target structures PauA7 and GlnA.
S2: Phenotypic analysis of parental strain and complemented glnA3 mutant on defined Evans-agar supplemented with polyamines.

## Slide 2
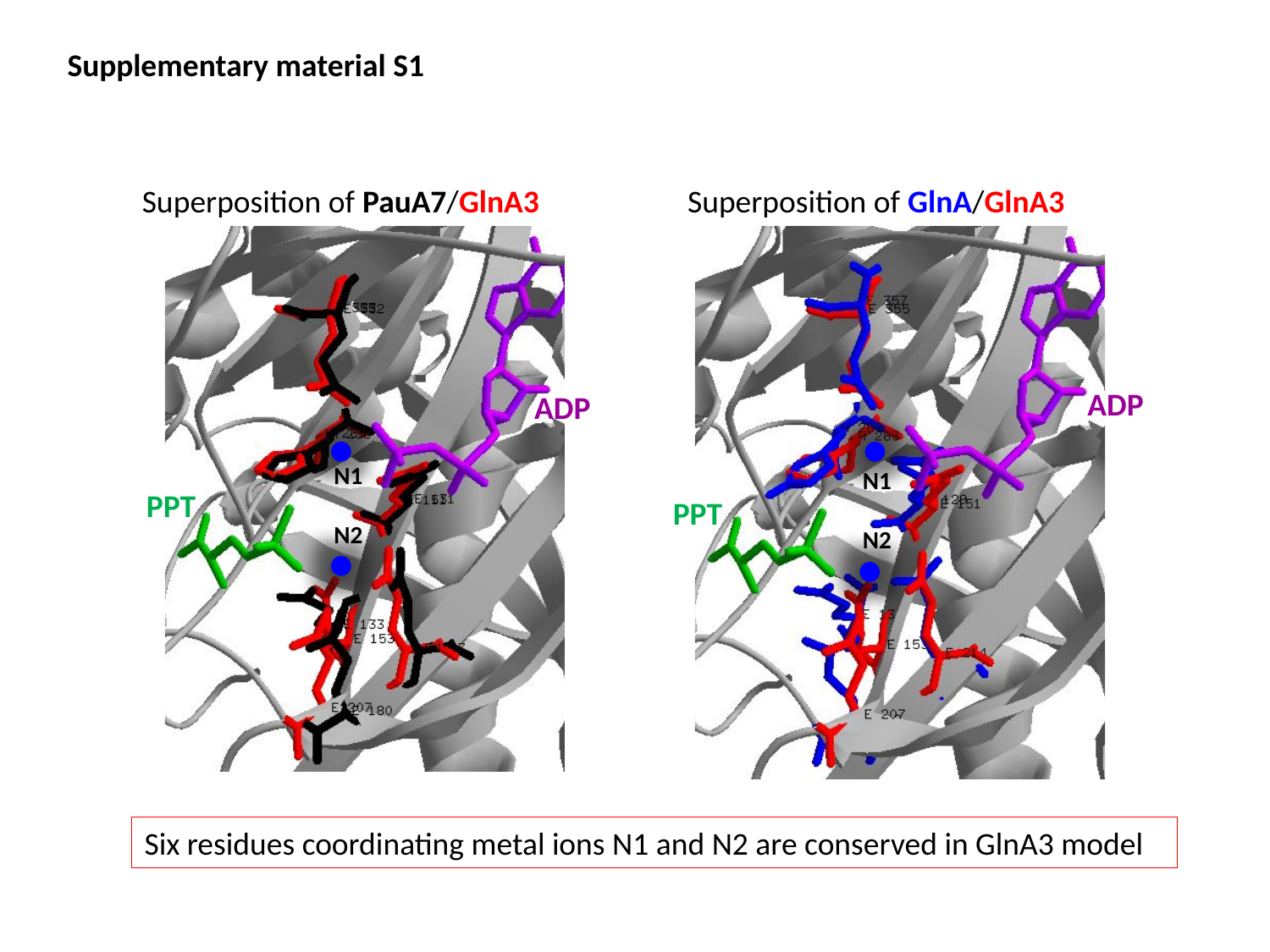

Supplementary material S1
Superposition of PauA7/GlnA3
Superposition of GlnA/GlnA3
ADP
ADP
N1
N2
N1
N2
PPT
PPT
Six residues coordinating metal ions N1 and N2 are conserved in GlnA3 model

## Slide 3
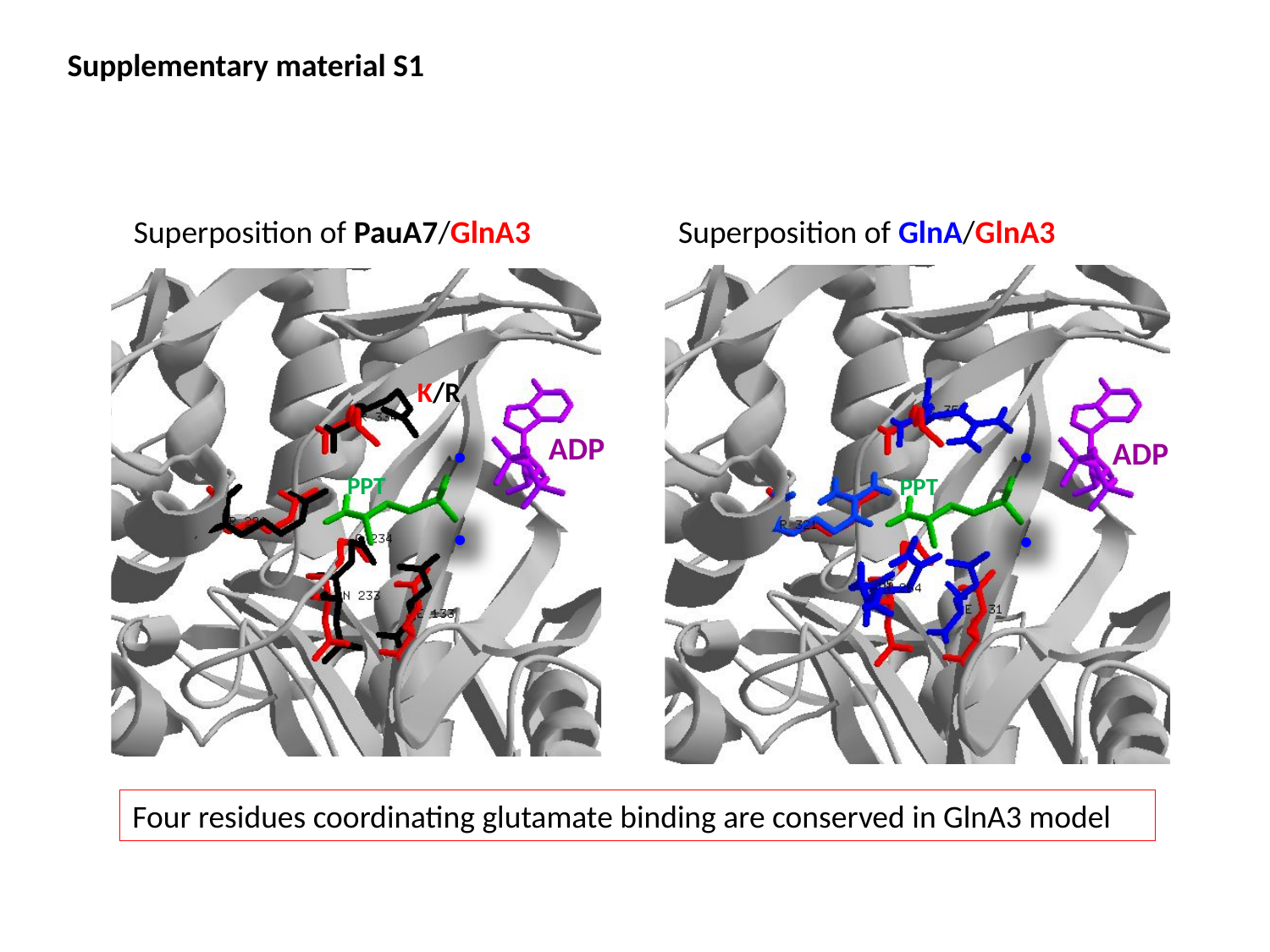

Supplementary material S1
Superposition of PauA7/GlnA3
Superposition of GlnA/GlnA3
K/R
ADP
ADP
PPT
PPT
Four residues coordinating glutamate binding are conserved in GlnA3 model

## Slide 4
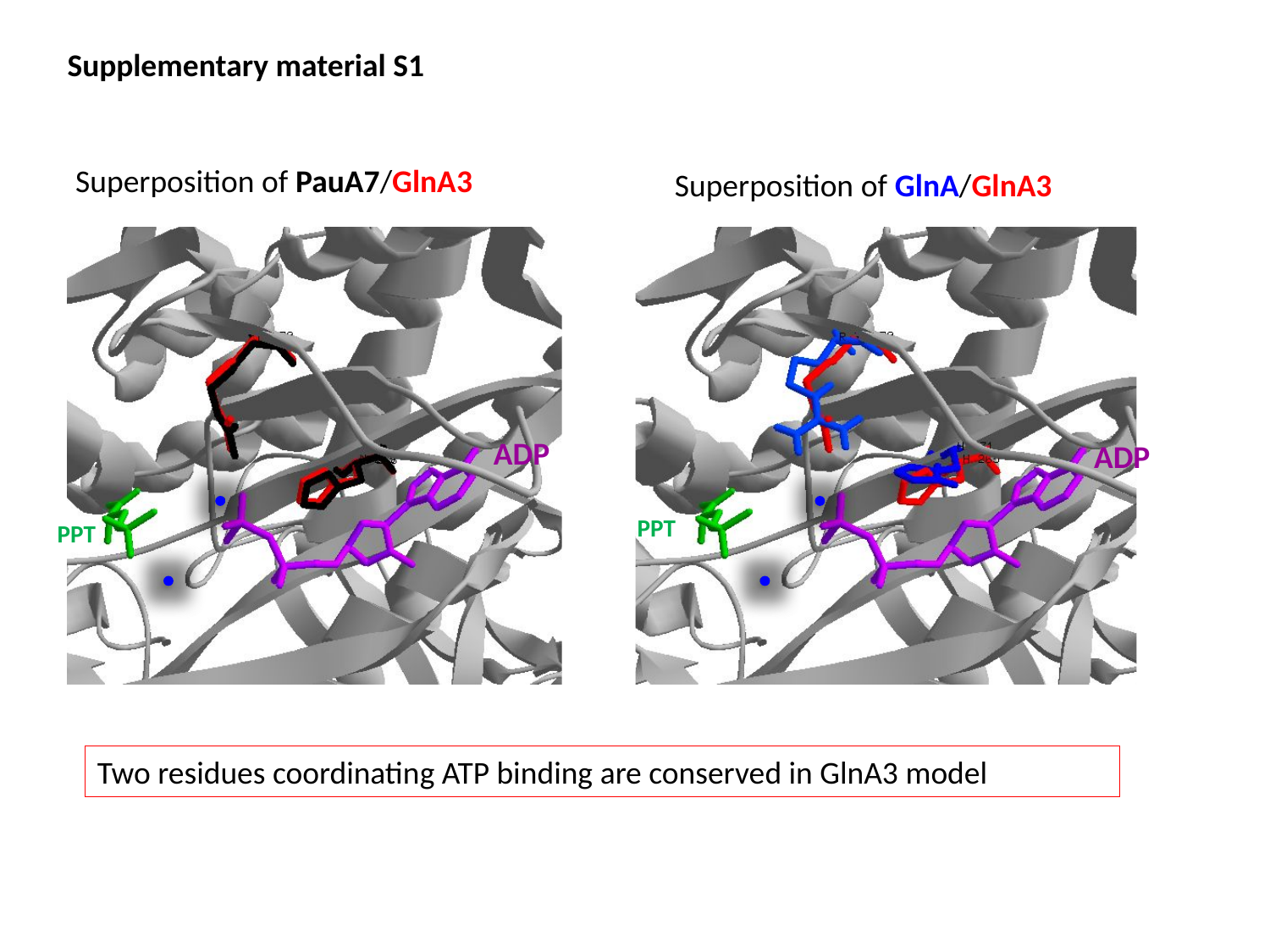

Supplementary material S1
Superposition of PauA7/GlnA3
Superposition of GlnA/GlnA3
ADP
ADP
PPT
PPT
Two residues coordinating ATP binding are conserved in GlnA3 model

## Slide 5
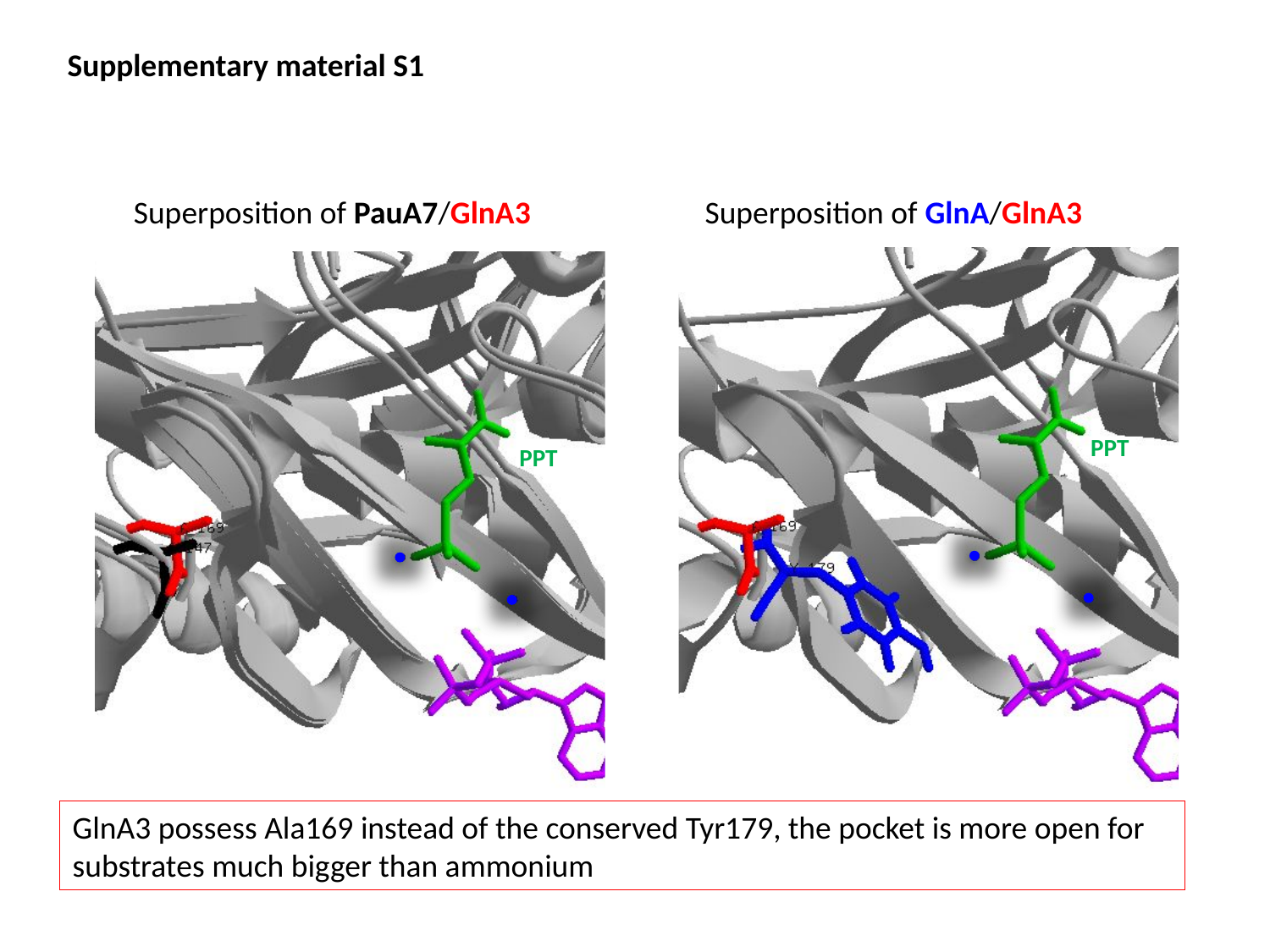

Supplementary material S1
Superposition of PauA7/GlnA3
Superposition of GlnA/GlnA3
PPT
PPT
GlnA3 possess Ala169 instead of the conserved Tyr179, the pocket is more open for substrates much bigger than ammonium

## Slide 6
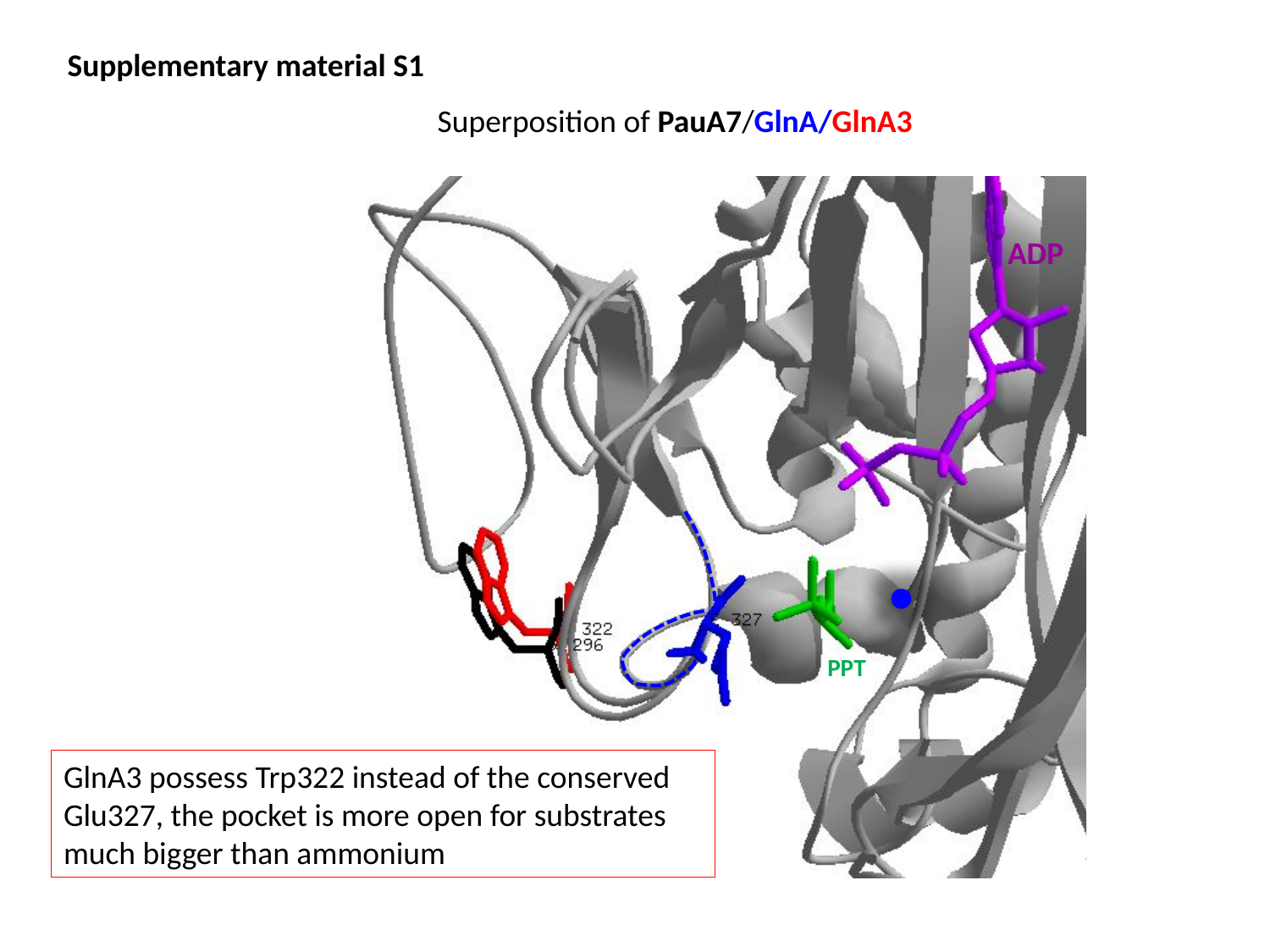

Supplementary material S1
Superposition of PauA7/GlnA/GlnA3
ADP
PPT
GlnA3 possess Trp322 instead of the conserved Glu327, the pocket is more open for substrates much bigger than ammonium

## Slide 7
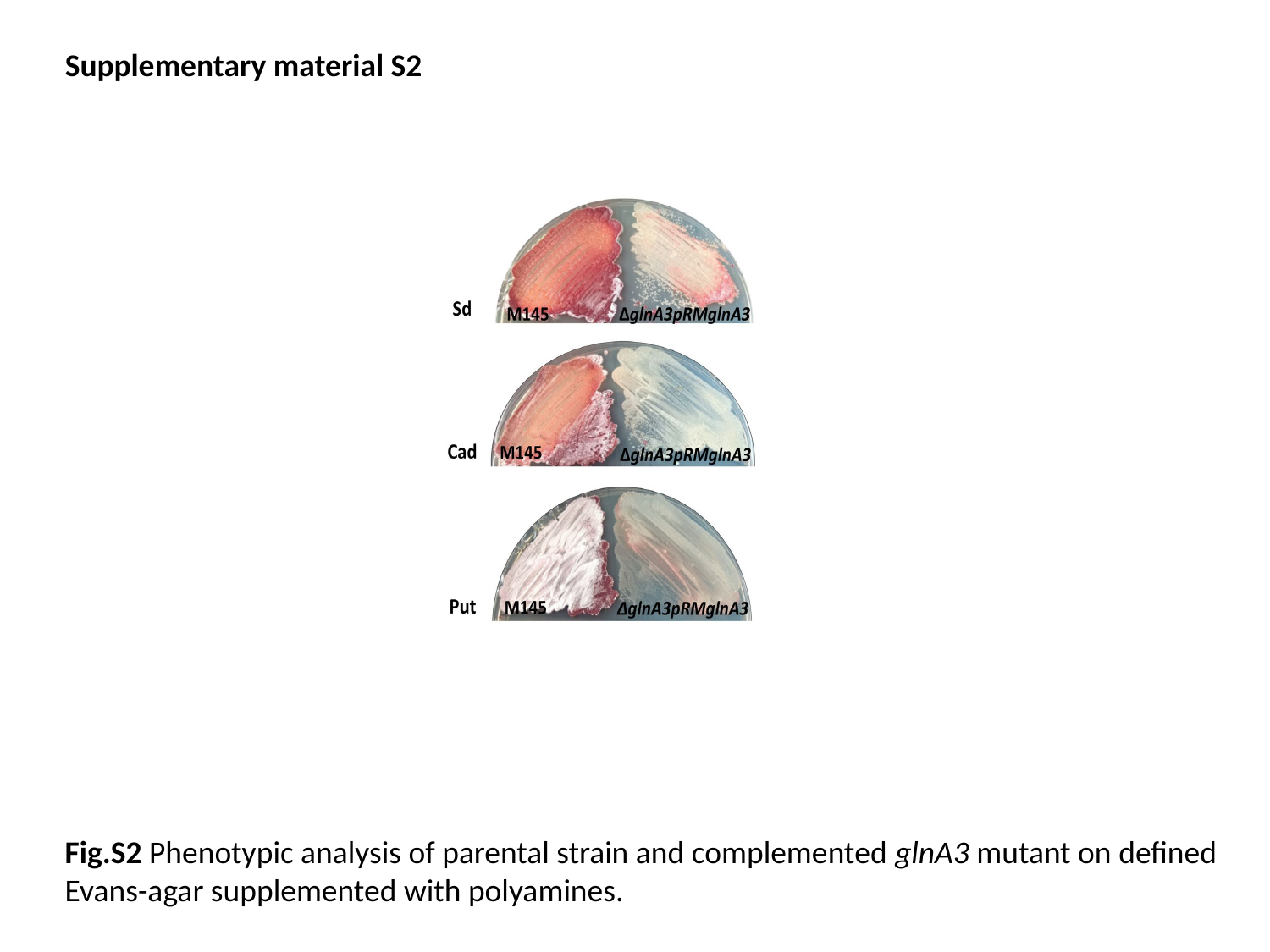

Supplementary material S2
Fig.S2 Phenotypic analysis of parental strain and complemented glnA3 mutant on defined Evans-agar supplemented with polyamines.
